# A Study of the Normal Movements of the Unimpregnated Uterus

**Published:** 1875-10

**Authors:** 


A STUDY OF THE NORMAL MOVEMENTS OF THE UNIMPREGNATED UTERUS. By Ely Van De Walker, M.D. Syracuse, N. Y. Pamphlet; pp. 26.

---

A STATEMENT OF THE RELATIONS of the Faculty of Medicine and Surgery in the University of Michigan to Homœopathy. Pamphlet; pp. 12.

